# Ubiquitin-speciﬁc protease 4 promotes metastasis of hepatocellular carcinoma by increasing TGF-β signaling-induced epithelial-mesenchymal transition

**DOI:** 10.18632/aging.101587

**Published:** 2018-10-18

**Authors:** Chan Qiu, Yan Liu, Ying Mei, Min Zou, Zhibo Zhao, Mingxin Ye, Xiaoling Wu

**Affiliations:** 1 Department of Gastroenterology, the Second Affiliated Hospital of Chongqing Medical University, Chongqing 400010, P.R. China; 2 Department of Gastroenterology, the Fifth People’s Hospital of Chengdu, ChengduSichuan, 611130, P.R. China; 3 Department of Hepatobiliary Surgery, the Second Affiliated Hospital of Chongqing Medical University, Chongqing 400010, P.R. China; 4 Department of Hepatobiliary Surgery, the Affiliated Hospital of Southwest Medical University, Lu zhou, Sichuan 646000, P.R. China; * Equal contribution

**Keywords:** hepatocellular carcinoma, Ubiquitin specific protease 4, metastasis, EMT, TGF-β

## Abstract

Invasion and metastasis are the main cause of recurrence and death in advanced hepatocellular carcinoma (HCC). Revealing the mechanisms of HCC metastasis is important for developing new therapeutic approaches and reducing patient mortality. Ubiquitin specific protease 4 (USP4), is involved in tumorigenesis by deubiquitinating some important oncogenic proteins and impacting their degradation. In the present study, we found that USP4 was significantly upregulated in HCC tumor tissues and the high expression of USP4 was associated with distant metastasis and poor survival in patients. Using gene interference, we demonstrated that USP4 knockdown significantly inhibited HCC cell migration and invasion *in vitro*, and USP4 overexpression had the opposite results. *In vivo*, we also found that USP4 knockdown obviously blocked HCC cell metastasis. Mechanistically, we revealed that USP4 interacted directly with and deubiquitinated TGF-β receptor type I (TGFR-1) to activate the TGF-β signaling pathway, and subsequently induced the Epithelial-Mesenchymal Transition (EMT) in HCC cells. Taken together, our results elucidate that USP4 is highly expressed in HCC and promotes the tumor invasion and metastasis, the underlying mechanism is that USP4 directly interacts with and deubiquitinates TGFR-1 to increase TGF-β signaling-Induced EMT. These results could provide a new therapeutic target for the treatment of HCC.

## Introduction

Hepatocellular carcinoma (HCC) is the most important pathological type of primary liver cancer, accounting for ~70–85% of all cases. It is the leading cause of death among patients with cirrhosis, and its incidence may continue to increase [1]. In Chinese men aged >60 years, the incidence and mortality of HCC are the highest [2]. Although many treatments for HCC, including surgical resection, liver transplantation, image-guided ablation, and molecular targeting therapy, have improved [3], these treatments are still unsatisfactory for advanced-stage HCC [4]. Invasion and metastasis are the main cause of postoperative recurrence and death of advanced HCC [5]. Therefore, revealing the mechanisms of HCC metastasis is important for developing new therapeutic approaches and reducing patient mortality.

The transforming growth factor-β (TGF-β) signaling pathway plays a critical role in regulating cell growth, differentiation, and development in a wide range of biological systems [6]. This signaling is initiated by ligand-induced oligomerization of serine/threonine receptor kinases type II receptors, which recruit and phosphorylate type I receptors. The activated type I receptors phosphorylate the cytoplasmic signaling molecules Smad2 and Smad3, which leads them to their partner with the common signaling transducer Smad4 and translocate to the nucleus. Activated Smad compounds then combine with transcription factors to exert diverse biological effects [7]. Dysregulation of the TGF-β signaling pathway results in various tumors, including HCC [8,9].The TGF-β signaling pathway contributes to the entire HCC process, including hepatic fibrosis, carcinogenesis, proliferation, invasion, and metastasis [9,10]. In HCC, TGF-β signaling induces the epithelial-mesenchymal transition (EMT) to promote HCC cell invasion and metastasis [11,12]. EMT is a reversible process in which epithelial cells change into mesenchymal cells with an altered cell adhesion and migration ability caused by the loss of the epithelial biomarker E-cadherin and an increase in mesenchymal markers such as N-cadherin and vimentin [13].

Deubiquitination is the reverse process of ubiquitination. It is catalyzed by deubiquitinating enzymes (DUBs) and has received attention for its crucial regulatory role in tumorigenesis [14]. Ubiquitin-specific proteases (USPs) are the largest subfamily of DUBs, comprising more than 60 members. USPs inhibit the degradation of target proteins that depend on the ubiquitin proteasome pathway and also regulate the location and activity of target proteins [15]. Accumulating evidence has confirmed that USPs are involved in tumorigenesis and malignancy by regulating key oncoproteins [16]. For example, USP2 regulates the stability of cyclin D1 to affect the cell cycle in tumor cells [17]. Silencing the expression of USP7 in wild-type p53 cells inhibits cell proliferation, whereas USP7 silencing can increase p53 levels by promoting the degradation of murine double minute2 (MDM2) [18]. USP4, a member of the USP family, is also closely related to tumor progression. It can activate the WNT/β-catenin signaling pathway by increasing the stability of β-catenin and promoting its accumulation in the nucleus [19]. USP4 interacts directly with and deubiquitinates ARF-BP1, leading to the stabilization of ARF-BP1 and subsequent reduction of p53 levels [20]. USP4 can also regulate the NF-κB signaling pathway by deubiquitinating and stabilizing HDAC2 [21]. These data suggest that USP4 can regulate various oncoproteins and signaling pathways to promote cancer progression. However, the regulatory roles and mechanisms of USP4 in HCC have not yet been elucidated. The purpose of this study was to assess the biological function and underlying mechanism of USP4 in the progression of HCC and provide a novel potential therapeutic target for the treatment of advanced HCC.

## RESULTS

### USP4 expression was significantly upregulated in HCC

**Figure 1:**
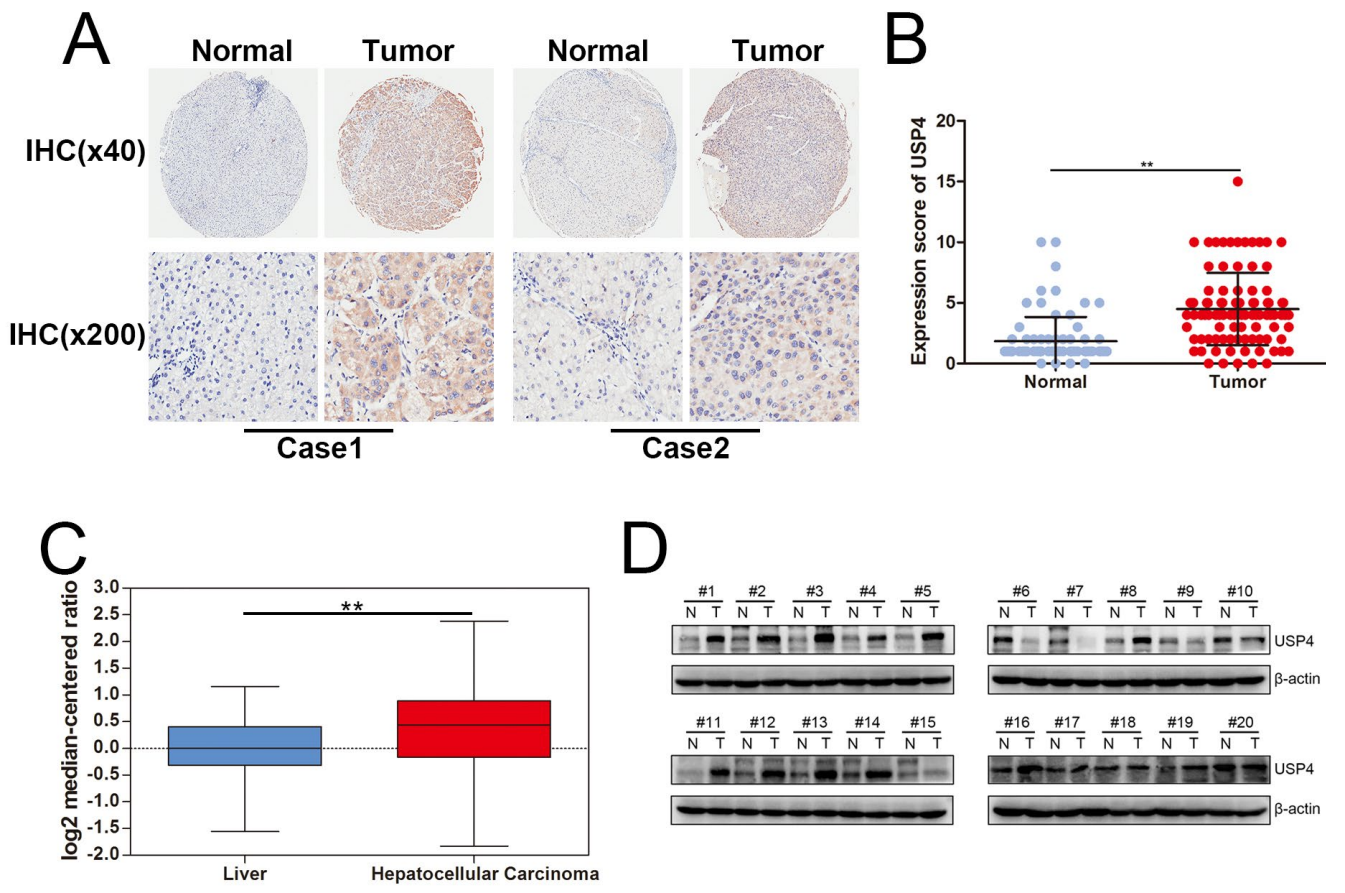
**USP4 expression was significantly upregulated in HCC.** (**A**) USP4 expression in HCC tumor tissues and matched surrounding tissues were examined by immunohistochemical staining. (magnification, ×40 and ×200). (**B**) Immunohistochemical scores of USP4 expression in HCC tumor tissues and matched surrounding tissues (** P < 0.01). (**C**) The mRNA level of USP4 in normal liver tissues and HCC tumor tissues which were collected from Oncomine data base (** P < 0.01). (**D**) USP4 expression in fresh HCC tumor tissues and matched surrounding tissue were examined by western blotting (N, matched surrounding tissues, T, tumor tissues).

To elucidate the regulatory roles of USP4 in HCC, tissue microarrays were first used to examine the expression of USP4 in HCC tissues. Immunohistochemical staining showed that USP4 was significantly upregulated in tumor tissues compared with matched surrounding tissues (Figs. 1A and B). Furthermore, we used Oncomine (https://www.oncomine.org/) to analyze the expression of USP4 and found that *USP4* mRNA levels were significantly higher in HCC tissues than in normal liver tissues (Fig. 1C). Finally, total proteins were extracted from fresh HCC tissues and matched surrounding tissues, and western blots confirmed that USP4 was overexpressed in tumor tissues compared with matched surrounding tissues (14/20=70%) (Fig. 1D). These results suggest that USP4 expression was significantly upregulated in HCC.

### Elevated USP4 expression was associated with HCC distant metastasis and poor survival

**Figure 2:**
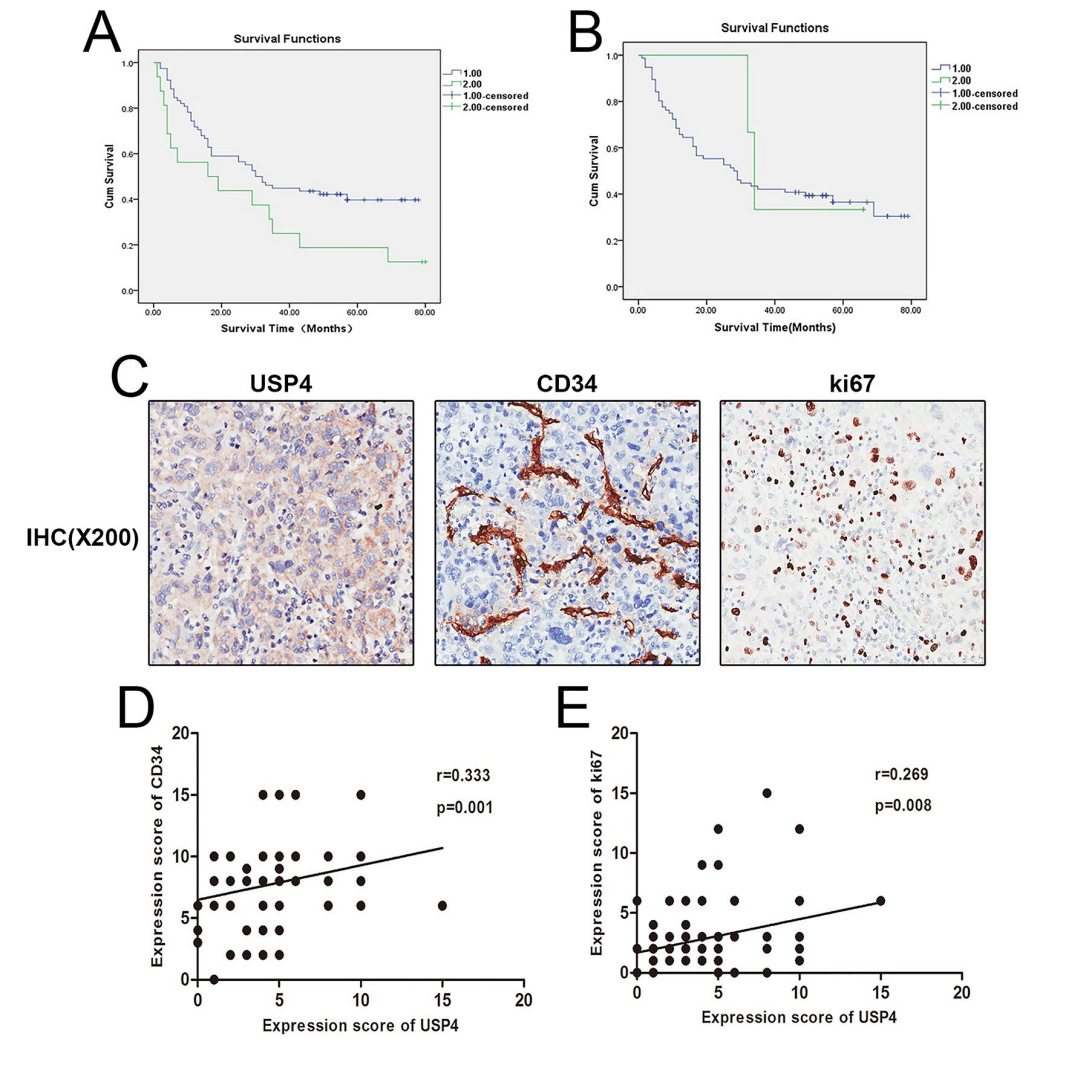
**High expression of USP4 was positive associated with HCC poor survival and some pathological characteristics.** (**A**) Kaplan–Meier survival curve showed the correlation between USP4 expression in tumor tissues and survival of patients with HCC, (P=0.032), 1: low USP4 expression, 2: high USP4 expression. (**B**) Kaplan–Meier survival curve shows the correlation between USP4 expression in matched surrounding tissues and survival of patients with HCC, (P=0.708), 1: low USP4 expression, 2: high USP4 expression. (**C**) USP4, Ki67 and CD34 expression were tested by immunohistochemical staining in HCC tissues (magnification, ×200). (**D**) USP4 expression was positively correlated with the expression of Ki67(p=0.001, r=0.333). (**E**) USP4 expression was positively correlated with the expression of CD34 (p=0.008, r=0.269).

The correlation between USP4 expression and the clinicopathological characteristics of HCC patients was analyzed using Spearman’s tests. The correlation analysis revealed that USP4 expression was positively associated with distant metastasis, but there was no significant correlation between USP4 expression and other clinicopathological features such as patient gender, age, and clinical stage (Table 1). Next, Kaplan-Meier analysis presented that in tumor tissues, but not surrounding tissues, high expression of USP4 was significantly associated with a lower survival rate (Figs. 2A and B). In addition, the multivariate analysis by Cox regression showed that USP4 expression was the only independent prognostic factor (Table 2). Besides, USP4 expression was positively correlated with the expression of Ki67 (Tumor proliferation marker) and CD34 (microvessel marker) (Figs. 2 C–E). These results demonstrated that USP4 expression was upregulated in HCC tumor tissues and was significantly associated with distant metastasis and poor patient survival. This suggests that USP4 may play an important role in the progression of HCC.

**Table 1:** The correlation between USP4 expression and clinicopathological characteristics of HCC patients.

			**Sex**	**Age**	**Size**	**Pathology grading**	**T**	**N**	**M**	**Clinical stage**
**Spearman's rho**	**Expression** **of USP4** **in tumor tissues**	**Correlation Coefficient**	**-.107**	**.147**	**-.096**	**.095**	**-.025**	**-.009**	**.249**	**-.038**
		**Sig. (2-tailed)**	**.305**	**.159**	**359**	**.363**	**.819**	**.935**	.021*	**.728**
		**N**	**94**	**93**	**93**	**94**	**86**	**85**	**86**	**85**
	**Expression** **of USP4** **in matched surrounding tissues**	**Correlation Coefficient**	**-.038**	**.116**	**.070**	**.072**	**.097**	**.134**	**-.065**	**.137**
		**Sig. (2-tailed)**	**.742**	**.308**	**.540**	**.528**	**.419**	**.269**	**.590**	**.257**
		**N**	**79**	**79**	**79**	**79**	**71**	**70**	**71**	**70**

* Correlation is significant at the 0.05 level (2-tailed).

**Table 2:** Multivariate analysis by Cox regression.

	**B**	**SE**	**Wald**	**df**	**P-Malue**	**Exp(B)**	**95.0% CI for Exp(B)** **Lower Upper**	

**Tumor size**	**.236**	**.367**	**.414**	**1**	**.520**	**1.267**	**.616**	**2.603**
**T**	**.342**	**.784**	**.190**	**1**	**.663**	**1.408**	**.303**	**6.546**
**Clinical stage**	**.344**	**.785**	**.192**	**1**	**.661**	**1.411**	**.303**	**6.572**
**USP4 expression**	**.845**	**.356**	**5.646**	**1**	.017*	**2.329**	**1.160**	**4.676**

* Correlation is significant at the 0.05 level (2-tailed).

### USP4 expression significantly impacted HCC cell migration and invasion *in vitro*

**Figure 3:**
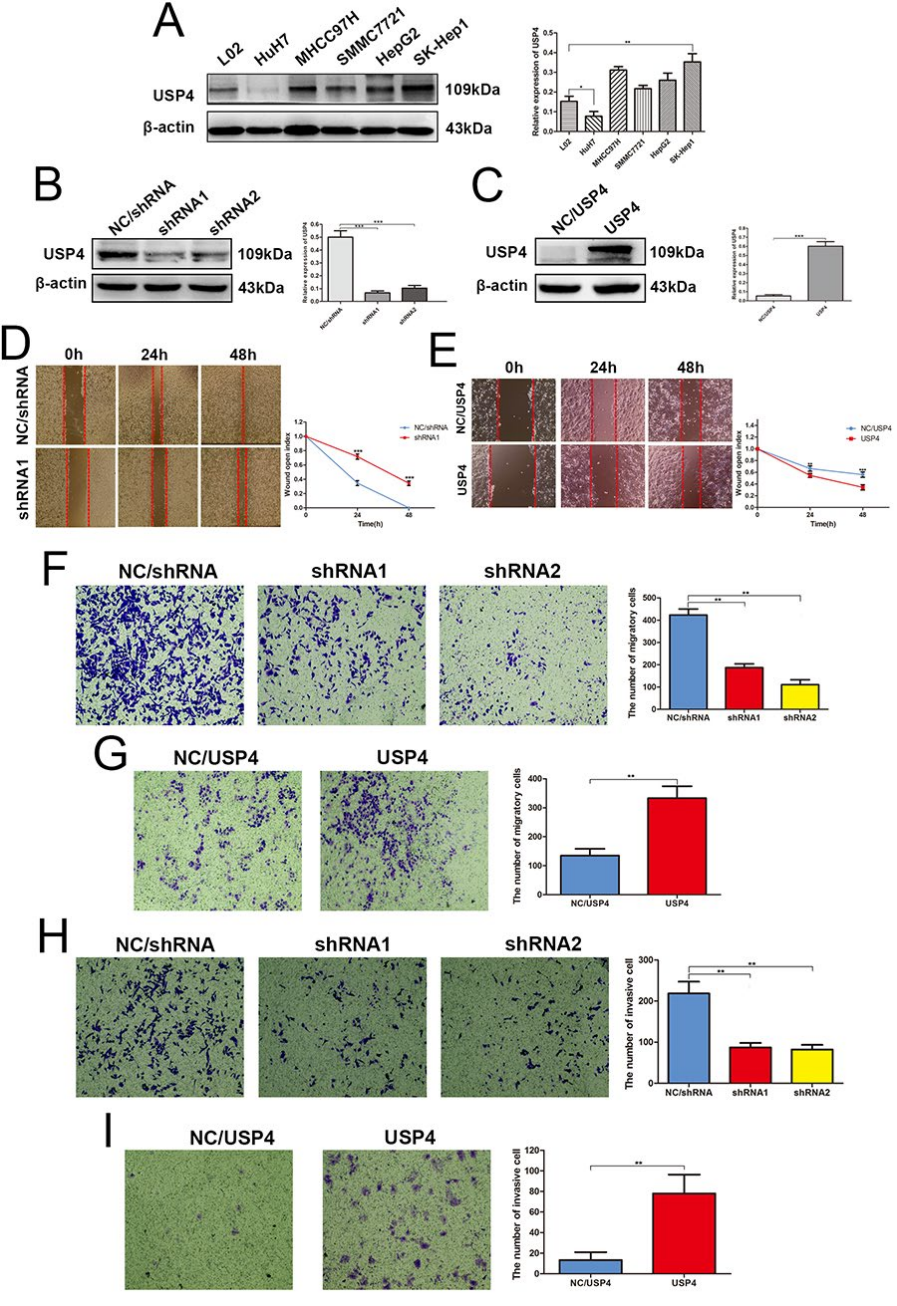
**USP4 expression significantly impacted HCC cell migration and invasion *in vitro.*** (**A**) USP4 expression was aberrant in HCC cell lines, as compared to the normal liver cell lines L02 (* P < 0.05, ** P < 0.01). (**B**) USP4 expression was knocked down by lentivirus technology in SK-Hep1cells (** P < 0.01). (**C**) USP4 was overexpressed by lentivirus technology in HuH7 cells (*** P < 0.001). (**D**) Wound-healing assays detected the effect of USP4 knockdown on the healing ability of SK-Hep1 cells (*** P < 0.001). (**E**) Wound-healing assays detected the effect of USP4 overexpression on the healing ability of HuH7 cells (** P < 0.01, *** P < 0.001). (**F**) Transwell assays evaluated the effect of USP4 knockdown on the migratory ability of SK-Hep1cells (** P < 0.01). (**G**) Transwell assays evaluated the effect of USP4 overexpression on the migratory ability of HuH7 cells (** P < 0.01). (**H**) Matrigel invasion assays examined the effect of USP4 knockdown on the invasive ability of SK-hep1 cells (** P < 0.01). (**I**) Matrigel invasion assays examined the effect of USP4 overexpression on the invasive ability of HuH7 cells (** P < 0.01).

Our previous results suggest that USP4 may play an important role in the progression of HCC. Thus, this hypothesis was assessed *in vitro* firstly. we detected the expression of USP4 in HCC cell lines using western blotting, and the results showed that USP4 expression was altered in HCC cell lines compared with human normal liver cell lines (Fig. 3A). Specifically, its expression was upregulated in SK-Hep1, HepG2, SMMC-7721, and MHCC97H cells and downregulated in HuH7 cells. Next, we used lentivirus technology to knock down USP4 expression in SK-Hep1 cells, which express high levels of endogenous USP4 (Fig. 3B), and overexpress USP4 in HuH7 cells, which express low levels of endogenous USP4 (Fig. 3C). These infected cells were treated with puromycin for 1 week to obtain stable cell lines and then used in subsequent experiments.

**Figure 4:**
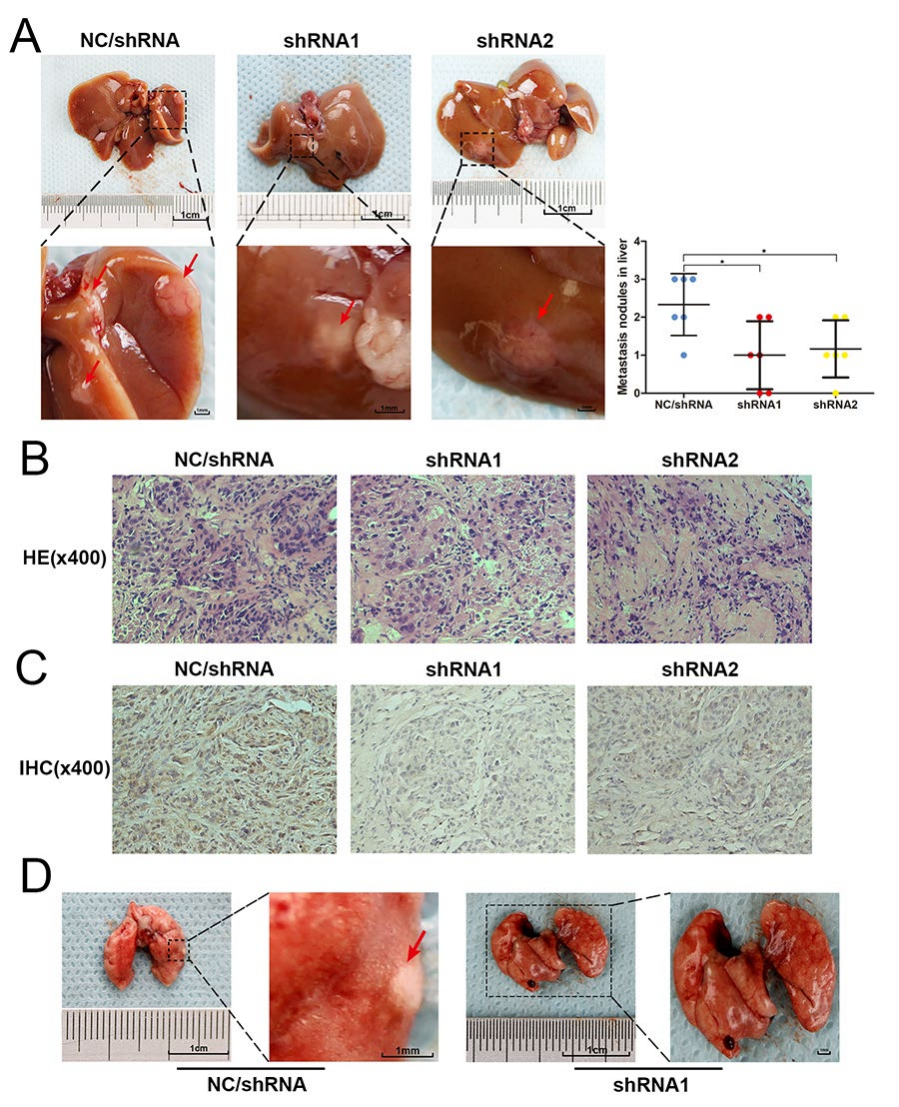
**USP4 knockdown blocked the metastasis of HCC cell *in vivo*.** (**A**) Representative images of hepatic metastatic nodules originated from SK-Hep1-shRNA/USP4 cells and negative control cells, and the quantitative analysis of hepatic metastatic nodules from each group (* P < 0.05). (**B**) The hematoxylin and eosin (HE) staining evaluated the pathological character of hepatic metastatic nodules originated from SK-Hep1-shRNA/USP4 cells and negative control cells (magnification ×400). (**C**) Immunohistochemical staining (IHC) examined the expression of USP4 in hepatic metastatic nodules originated from SK-Hep1-shRNA/USP4 cells and negative control cells (magnification ×400). (**D**) Representative images of metastatic nodules in lung originated from negative control cells.

Wound healing and Transwell migration assays were used to assess the effects of USP4 on HCC cell migration. Wound healing assays showed that the healing capability of SK-Hep1-shRNA/USP4 cells was significantly weakened compared with negative control cells (Fig. 3D). Whereas, the healing capability of HuH7-USP4 cells was significantly enhanced compared with negative control cells (Fig. 3E). Similarly, Transwell migration assays demonstrated that USP4 knockdown significantly reduced the migratory ability of SK-Hep1 cells (Fig. 3F), Whereas USP4 overexpression significantly enhanced this ability in HuH7 cells (Fig. 3G). Further, Matrigel invasion assays revealed a lower invasion ability in SK-Hep1-shRNA/USP4 cells compared with negative control cells (Fig. 4H), whereas a higher invasion ability was observed in USP4-overexpressing HuH7 cells (Fig. 4I). These data suggest that USP4 expression significantly impacted HCC cell migration and invasion *in vitro.*

### USP4 knockdown obviously blocked HCC cell metastasis *In vivo*.

To further investigate the role of USP4 on HCC cell metastasis, the effect of knocking down USP4 on tumor metastasis was assessed in an orthotopic metastatic mouse model. An equal number of SK-Hep1-shRNA/USP4 and negative control cells were injected into the liver of 4–6-week-old male BALB/c nude mice (n = 6/group). Eight weeks later, nude mice were sacrificed and the livers and lungs were separated. Metastatic tumors in the liver were investigated, which demonstrated that SK-Hep1-shRNA/USP4 cells produced fewer and smaller hepatic metastatic nodules than negative control cells (Figs. 4A). Then, metastatic nodules embedded in and sectioned for histopathological analysis. Hematoxylin and eosin (H&E) staining confirmed that the nodules were tumors (Fig. 4B). Immunohistochemical (IHC) staining demonstrated USP4 expression in these nodules and confirmed that USP4 expression was much lower in the nodules derived from SK-Hep1-shRNA/USP4 cells compared with negative control cells (Fig. 4C). Noteworthy, some metastatic tumors were also found in the lungs of some mice in the negative control group. However, no metastatic tumors were found in the SK-Hep1-shRNA/USP4 cell group (Fig. 4D).

In summary, these results demonstrated that USP4 expression contributes to HCC cell invasion and metastasis, suggesting that USP4 functions as a tumor promoter in HCC.

### USP4 activated the TGF-β signaling pathway to induce EMT in HCC cell

**Figure 5:**
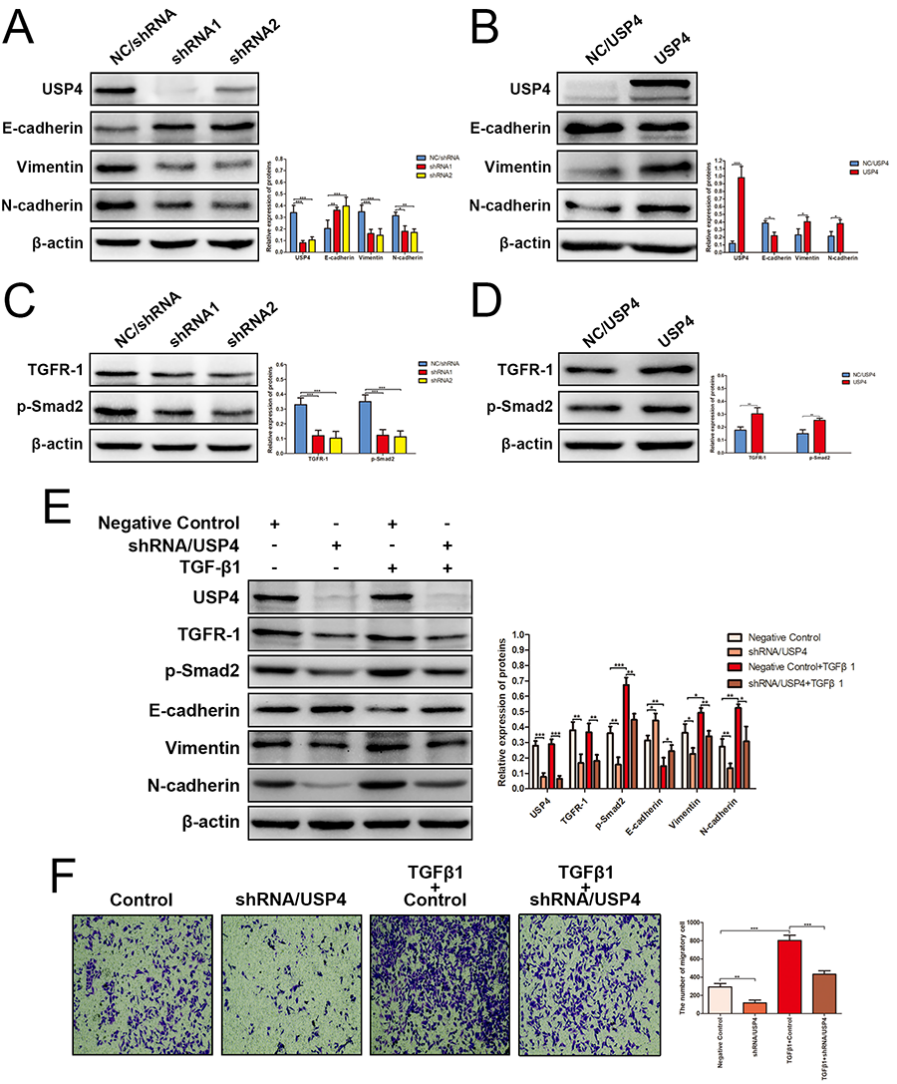
**USP4 activated TGF-β signaling pathway to induce epithelial-to-mesenchymal transition (EMT).** (**A**) The expression of EMT markers in SK-Hep1- shRNA/USP4 cells and in negative control cells (* P < 0.05, ** P < 0.01, *** P < 0.001). (**B**) The expression of EMT markers in HuH7-USP4 cells and in negative control cells (* P < 0.05, *** P < 0.001). (**C**) The expression of TGFR-1 and p-Smad2 in SK-Hep1- shRNA/USP4 cells and in negative control cells (*** P < 0.001). (**D**) The expression of TGFR-1 and p-Smad2 in HuH7-USP4 cells and in negative control cells (* P < 0.05, ** P < 0.01). (**E**) The expression of TGFR-1, p-Smad2 and EMT markers in SK-Hep1- shRNA/USP4 cells or in negative control cells, with TGF-β1(10ng/mL)treatment for 24h or without (* P < 0.05, ** P < 0.01, *** P < 0.001). (**F**) The migration ability of SK-Hep1-shRNA/USP4 cells or negative control cells, with TGF-β1 treatment or without, detected by Transwell migration assay (** P < 0.01, *** P < 0.001).

The EMT is a reversible process in which epithelial cells change into mesenchymal cells; it is a crucial driver of cancer migration and invasion [13]. To reveal the mechanism by which USP4 promotes HCC metastasis, we next assessed the effect of USP4 on EMT progression. The results presented that knocking down USP4 obviously reversed EMT process (Fig. 5A), whereas USP4 overexpression promoted EMT progression (Fig. 5B). These results suggest that USP4 promoted HCC cell invasion and metastasis, probably by inducing EMT. However, many upstream elements and signaling pathways regulate EMT [22]. Among these, the TGF-β signaling pathway is the most reported [23,24]. TGF-β receptor activation leads to Smad2 and Smad3 phosphorylation, and Smad2 phosphorylation is frequently used to assess the activation status of the TGF-β signaling pathway [25]. Here, we measured the levels of TGF-β receptor type I (TGFR-1) and p-Smad2 in response to altered USP4 expression. The expression of TGFR-1 and p-Smad2 was significantly decreased when USP4 was knocked down (Fig. 5C), whereas overexpressing USP4 increased the expression of TGFR-1 and p-Smad2 (Fig. 5D), which indicating that USP4 could activated TGF-β signaling. Taken together, the above results suggested that USP4 could activate TGF-β signaling pathway to induce EMT process. To confirm this finding, we treated SK-Hep1-NC/shRNA cells and SK-Hep1-shRNA/USP4 cells with TGF-β1 (a agonist of TGF-β signaling pathway) (StemRD, CA, USA). After 24 hours of treatment, total proteins were extracted and western blotting was performed to examine the status of the TGF-β signaling pathway and EMT markers. The results showed that treatment with TGF-β1 agonist strongly activated the TGF-β signaling pathway and induced EMT process. However, this promoting effect was significantly impaired by knocking down USP4 expression (Fig. 5E). Finally, migration assays demonstrated that treatment with a TGF-β1 agonist enhanced the migratory ability of HCC cells, whereas USP4 knockdown inhibited this effect (Figs. 5F).

### USP4 directly interacted with and deubiquitinated TGFR-1 in HCC cell

**Figure 6:**
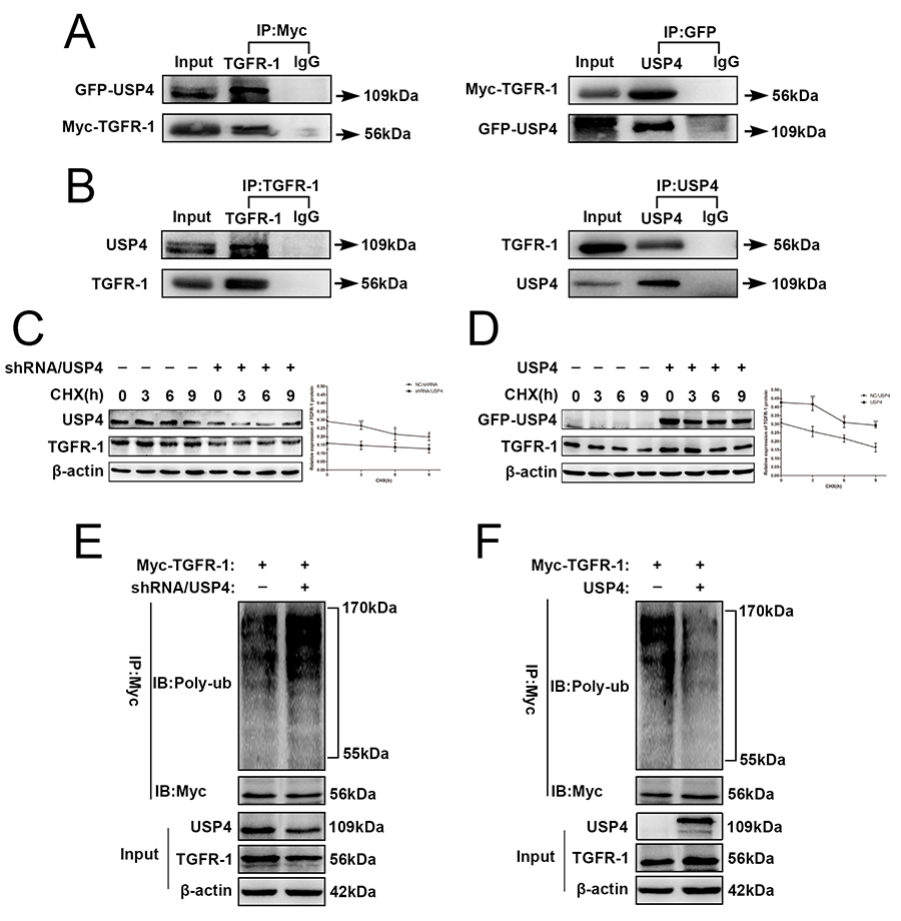
**USP4 directly interacted with and deubiquitinated TGFR-1 in HCC cell.** (**A**) USP4 interacted with TGFR-1 at exogenous levels. Immunoblotting analysis of lysates after immunoprecipitation from SK-Hep1 cells co-transfected with GFP-USP4 and Myc-TGFR-1. IgG was used as a negative control. (**B**) USP4 interacted with TGFR-1 at endogenous levels. Cell lysates from wild type SK-Hep1cells were immunoprecipitated with anti-USP4 or anti- TGFR-1 antibody, followed by immunoblotting with anti- TGFR-1 or anti-USP4 antibody, respectively. IgG was used as a negative control. (**C**) USP4 knockdown reduced the stability of TGFR-1 protein (* P < 0.05, *** P < 0.001). (**D**) USP4 overexpression elevated the stability of TGFR-1 protein (** P < 0.01, *** P < 0.001). SK-Hep1-shRNA/USP4 cells or SK-Hep1-USP4 cells were treated with protein synthesis inhibitor CHX (100 µg/mL) for the indicated times. Then, proteins were extracted and Western blotting was performed. (**E**) Effect of USP4 knockdown on the polyubiquitin levels of TGFR-1. (**F**) Effect of USP4 overexpression on the polyubiquitin levels of TGFR-1. SK-Hep1-TGFR-1 cells were co-infected with shRNA/USP4 or USP4. 48h later, cells were treated with proteasome inhibitor MG132 for 6 hours. Then, TGFR-1 was immunoprecipitated with anti-Myc antibody, and the polyubiquitination of TGFR-1 was detected by immunoblotting.

The data described above indicate that USP4 activates the TGF-β signaling pathway to induce the EMT in HCC cells. Importantly, a previous study reported that TGFR-1 is regulated by ubiquitination [26]. Therefore, USP4 may directly interact with TGFR-1 to activate TGF-β signaling in HCC cells. GFP-USP4 and Myc- TGFR-1 overexpression lentiviruses were co-infected into SK-Hep1 cells. Forty-eight hours later, total proteins were extracted and used to detect binding between exogenous USP4 and TGFR-1. Co-immunoprecipitation assay showed that exogenous USP4 and TGFR-1 directly bound to each other (Fig. 6A). Then, total proteins were extracted from wild-type SK-Hep1 cells and the co-immunoprecipitation assay identified the binding between USP4 and TGFR-1, suggesting that there was also a direct interaction between these proteins endogenously (Fig. 6B).

USP4, a member of the USP family, inhibits the degradation of target proteins that depend on the ubiquitin proteasome pathway. Therefore, we assessed the effect of USP4 expression on the stability of endogenous TGFR-1 protein, cycloheximide (CHX) chase assay presented that the half-life of TGFR-1 protein in SK-Hep1 cells was decreased after knockdown of USP4 (Fig. 6C). However, overexpression of USP4 in SK-Hep1 cells elevated the half-life of TGFR-1 (Fig. 6D). This evidence indicated USP4 stabilizes TGFR-1 protein expression in HCC cell.

Sequentially, we monitored the effect of USP4 on TGFR-1 ubiquitination in SK-Hep1 cells. Co-immunoprecipitation assays demonstrated that TGFR-1 ubiquitin levels were increased significantly in USP4 knock down compared with negative control cells (Fig. 6E), whereas USP4 overexpression yielded the opposite results (Fig. 6F). These data demonstrated that USP4 interacts directly with and deubiquitinates TGFR-1 in HCC cell.

### USP4 expression was positively correlated with EMT process in HCC tumor tissues

**Figure 7:**
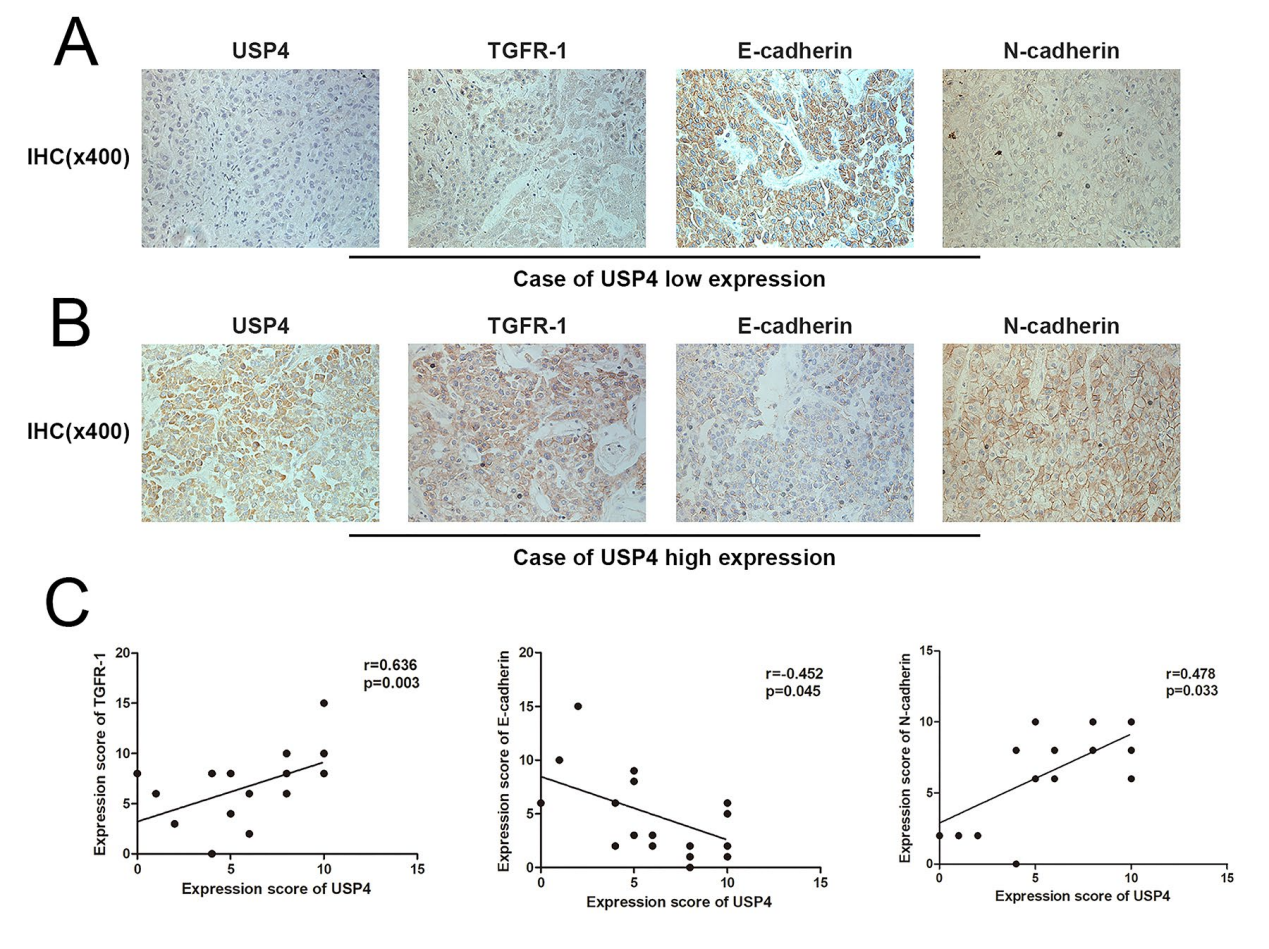
**USP4 expression was positively correlated with EMT process in HCC tumor tissues.** (**A**) Immunohistochemical staining (IHC) detected the expression of USP4, TGFR-1, E-cadherin, N-cadherin in USP4 low expression cases (magnification, ×400). (**B**) Immunohistochemical staining (IHC) detected the expression of USP4, TGFR-1, E-cadherin, N-cadherin in USP4 high expression cases (magnification, ×400). (**C**) The correlation between USP4 expression and TGFR-1, E-cadherin, N-cadherin was analyzed by Spearman rank correlation. r>0 and p < 0.05 was considered positive associated, r<0 and p < 0.05 was considered negative associated.

It's exciting that we revealed that USP4 activates the TGF-β signaling pathway to induce EMT in HCC cell. However, the finding was just obtained from cellular or molecular level, it needs to further identification in clinical samples. Therefore, we performed IHC assays to detect the expression of USP4, TGFR-1 and EMT markers in HCC tumor tissues and analyze their correlation. As we expected, in USP4 low expression cases, TGFR-1 and N-cadherin presented low expression and E-cadherin presented high expression (Fig. 7A), whereas in USP4 high expression cases, TGFR-1 and N-cadherin presented high expression and E-cadherin presented low expression (Fig. 7B). finally, we performed correlation analysis to evaluate their correlation and found that USP4 expression was positive associated with TGFR-1 and N-cadherin expression, and was negative associated with E-cadherin expression (Fig. 7C). The evidence strongly confirms our findings on cellular level.

## DISCUSSION

With the regulatory role of deubiquitination in tumorigenesis is high valued, a key role for the deubiquitinase USP4 has been reported in some cancers. A recent study confirmed that upregulated USP4 plays an oncogenic role in melanoma [27]. In another study, USP4 controlled metastatic potential by stabilizing β-catenin in brain metastatic lung adenocarcinoma [28]. USP4 also positively regulated WNT/β-catenin signaling in colorectal cancer [19]. However, the expression status and role of USP4 in HCC has not been reported extensively. In the present study, tissue microarrays showed that USP4 was significantly upregulated in HCC tumor tissues compared with matched surrounding tissues. Furthermore, the high expression of USP4 was positively associated with HCC distant metastasis and poor survival. Biological function experiments revealed that USP4 knockdown inhibited HCC cell migration and invasion, whereas USP4 overexpression had the opposite effect. Knocking down USP4 significantly reduced intrahepatic and pulmonary metastasis *in vivo*. These results suggest that USP4 is an oncogene in HCC, which is consistent with findings in other cancers. Studies have reported that USP4 could inhibit breast cancer cell growth by upregulating PDCD4 [29]. In addition, USP4 plays a tumor suppressor role in head and neck squamous cell carcinoma (HNSCC) by negatively regulating RIP1-mediated NF-κB activation and promoting TNF-α-induced apoptosis [30]. These findings suggest that USP4 could be a tumor suppressor in some cancers. The main reason for the distinct roles of USP4 in cancers could be that USP4 has different partners in different tumor types. Interestingly, even in the same cancer and signaling pathway, USP4 presents opposite mediation mechanism and role. For example, an early study revealed that USP4 interacts with Nlk, a negative regulator of Wnt signaling, to block the Wnt signaling pathway [31], and a later report demonstrated that USP4 acts as a positive regulator of the WNT/β-catenin pathway by deubiquitinating and facilitating the nuclear localization of β-catenin [19].

In the current study, we also assessed the effect of USP4 on HCC cell proliferation, and the results indicated that USP4 promoted HCC cell proliferation (data not shown). We focused on the effects of USP4 on HCC cell metastasis in the present study for two reasons. First, tissue microarray data demonstrated that high USP4 expression was positively associated with distant metastasis and poor survival in HCC patients, suggesting that USP4 plays a more important role in HCC invasion and metastasis than in tumor cell proliferation. Second, invasion and metastasis are the main cause of postoperative recurrence and death in advanced HCC patients, and revealing the mechanisms of HCC metastasis is important for developing new therapeutic approaches and reducing patient mortality.

The EMT is a complex process in which epithelial cells develop a mesenchymal phenotype; it plays a vital role in the early stage of tumor cell metastasis as cells lose intercellular cohesion and use an increased migratory and invasion ability to spread into distant tissues [32]. The role of the EMT in facilitating HCC cell invasion and metastasis is widely accepted [11]. The current study found that USP4 could promote HCC cell invasion and metastasis *in vivo* and *in vitro*. Therefore, we assessed the effect of USP4 on EMT progression. The results revealed that knocking down USP4 reversed the EMT whereas USP4 overexpression promoted the EMT, suggesting that USP4 promotes HCC cell migration and invasion by inducing the EMT. The ability of the TGF-β signaling pathway to induce the EMT to promote HCC cell invasion and metastasis is well-studied. The current results demonstrated that USP4 interacted directly with and deubiquitinated TGFR-1 and activated the TGF-β signaling pathway to induce the EMT, which was also identified in HCC clinical samples. Previous studies have shown that USP4 deubiquitinates and regulates TGFR-1 in other tumor types [33]. As we mentioned before, USP4 has different partners in different tumor types, which results in distinct functions. Therefore, we put forward a point that USP4 promotes HCC metastasis by increasing TGF-β signaling-induced EMT, and the USP4-TGF-β-EMT signal axis is first complete identified in HCC.

The ubiquitin-proteasome system (UPS) has developed as a therapeutic target for the treatment of cancers. For example, the UPS inhibitors bortezomib and lenalidomide have been applied to hematologic malignancies [34]. Other specific inhibitors targeting ub-activating enzyme (E1), ub-conjugating enzyme (E2), and ub-ligating enzyme (E3) are also considered valid therapeutic strategies, and many targets are currently being investigated. Another promising approach toward regulating UPS is to target deubiquitinases (DUBs) [35]. USPs, which are a subgroup of DUBs, have attracted attention as cancer targets because of their dysregulated expression and tumor-promoting effects. USPs have some advantages that make them ideal candidates for drug development. For example, they reverse the ubiquitination of specific substrate proteins by hydrolyzing the isopeptide or a-peptide bonds that link ubiquitin to target proteins, which may avoid the deleterious side effects associated with targeting the proteasome directly [36,37]. Besides, each USP has at least one or more non- conservative catalytic domains making them have their individual substrates, it could be advantageous to target the interaction domains of individual substrate protein, which contributes to target unique substrate recognition and avoid cross-reactivity [14]. In this study, the finding that USP4 has a crucial regulatory role in HCC metastasis may provide new clues for advanced HCC treatment.

In summary, the present study demonstrated that USP4 contributes to HCC invasion and metastasis by directly interacting with and deubiquitinating TGFR-1 to activate the TGF-β signaling pathway and induce the EMT. This study may highlight a new therapeutic opportunity for treating advanced HCC by targeting USP4.

## MATERIALS AND METHODS

### Main reagents

Antibodies against USP4, Ki67 CD34 and TGFR-1 were purchased from Abcam (Cambridge, UK). Antibodies against E-cadherin, Vimentin, E-cadherin, p-smad2, GFP-Tag, Myc-Tag, K48-linkage specific polyubiquitin were purchased from Cell Signaling Technology (CST, Danvers, MA, USA). Cycloheximide (CHX) was purchased from Abmole (Shanghai, China), MG132 was purchased from MCE (Monmouth Junction, NJ, USA).

### Tissue microarrays and tissue samples

A tissue microarray including 95 HCC tissues and 85 matched surrounding tissues, collected between 2007 and 2009, was purchased from Shanghai Outdo Biotech (Shanghai, China). The clinicopathological characteristics of all patients, including gender, age, stage, tumor size, pathological type, and clinical stages, were obtained (Supplementary Table 1). All patients were followed up for 4–6 years.

Fresh HCC tissues and matched surrounding tissues were obtained from patients treated with primary surgery at the Second Affiliated Hospital Surgery Department of Chongqing Medical University (Chongqing, China). All samples were evaluated and subjected to histological diagnosis by pathologists. Clinicopathological characteristics were also obtained. All patients involved in this study provided informed consent that their tissues could be retained and analyzed for research purposes only. The study was approved by the Human Research Ethics Committee of the Second Affiliated Hospital of Chongqing Medical University.

### Cell culture

Human HCC cell lines HepG2 and HuH7 were obtained from the American Type Culture Collection (ATCC, VA, USA). The HCC cell lines SK-Hep1, SMMC-7721, and MHCC97H and the human normal liver cell line L02 were purchased from the Cell Bank of the Chinese Academy of Sciences (Shanghai, China). Cells were maintained in high glucose Dulbecco's Modified Eagle (DMEM) medium (Hyclone Laboratories, UT, USA) containing 10% fetal bovine serum (Gibco, NY, USA) in a humidified atmosphere at 37°C containing 5% CO_2_.

### Immunohistochemistry

A Biotin-Streptavidin HRP Detection System (ZSGB-BIO, Beijing, China) was used for immunohistochemical staining. Dewaxing and antigen retrieval was performed firstly. Then, 3% H_2_O_2_ was used to quench peroxidase activity. The microarray was blocked with normal goat serum at room temperature for 15 minutes and then incubated with primary antibody overnight at 4°C. The next day, lgG/Bio working solution was added and the array was incubated at room temperature for 15 minutes. Then, S-A/HRP (horse radish peroxidase) solution was added and incubated for 15 minutes at room temperature. Microarray was stained by the freshly formulated diaminobenzidine (DAB) work fluid appropriately and were washed with running water timely. Finally, the microarray was counterstained with hematoxylin and covered with coverslips.

The staining intensity was scored as follows: 0, no staining; 1, weak staining; 2, intermediate staining; and 3, strong staining. The positive rate score was determined as follows: 0, 0 of the cells stained positive; 1, 1%–20% of the cells stained positive; 2, 21%–40% of the cells stained positive; 3, 41%–60% of the cells stained positive; 4, 61%–80% of the cells stained positive; and 5, 81%–100% of the cells stained positive. The total score was the combination of the staining intensity and staining positive rate scores. Samples with a total score <6 and ≥6 were defined as the low and high expression groups, respectively. The expression of USP4, Ki67, and CD34 was scored according to this method.

### Lentivirus infection

Lentiviruses carrying small hairpin RNA (shRNA) and the overexpression sequence of *USP4* were synthesized by Genechem Company (Shanghai, China). The human USP4 shRNA-specific target sequences used are as follows: shRNA1, GGCGTGGAATAAACTACTA; shRNA2, AGAGTCAGACCTTGAAAGA; shRNA3, GAAACACGGCTCTGGAACA. Cells were planted into six-well plates and cultured overnight. They were then washed with PBS and 1-mL of medium was added. Then, the lentivirus was added directly to the medium (MOI=20). A total of 4–6 h later, the volume of medium was increased to 2 mL. Twenty-four hours later, the medium containing the lentivirus was discarded and replaced with new medium. After 48 h, puromycin was added to the medium for 1 week to select stably infected cells. Finally, total proteins were extracted from the stably infected cells to assess the efficiency of knockdown and overexpression.

### Wound-healing assays

Cells were seeded in six-well plates and incubated until they reached complete confluence. The confluent monolayers were wounded in a line using a sterile plastic pipette tip and washed with PBS twice to remove any detached cells. The distance migrated by cells across the wound was measured over a fixed time interval. For accuracy, at least three regions were selected for statistical analysis.

### Transwell migration assays

A total of 100 µL serum-free medium containing 2 × 10^4^ cells was added to the upper compartment of a 24-well Transwell™ Chamber (Corning, NY, USA) and the bottom chamber was filled with 700 µL complete medium. Twenty-four hours later, migratory cells were fixed with paraformaldehyde for 30 min, stained with crystal violet for 30 min, and photographed with inverted microscope. Cells were counted in three random fields to quantify migration.

### Matrigel invasion assays

For Matrigel invasion assays, Matrigel (BD Biosciences, San Diego, CA, USA) was diluted with serum-free medium according to the manufacturer’s instructions and added to the Transwell upper chamber, incubated at 37°C until the Matrigel solidified. Then, 100 µL serum-free medium containing 2 × 10^4^ cells was added to the upper chamber on top of the solidified Matrigel and the lower chamber was filled with 700 µL medium containing 10% FBS. After incubation for 48 h, the migratory cells were fixed with paraformaldehyde, stained with paraformaldehyde, and counted in three random fields.

### Orthotopic metastatic mouse model

To evaluate tumor metastasis *in vivo*, an orthotopic metastatic mouse model of liver cancer was used [38]. Briefly, 4–6-week-old male BALB/c nude mice were selected (SPF grade, n = 6 per group). A total of 5 × 10^7^ cells in the logarithmic phase were collected and maintained in 1 mL PBS. Mice were anesthetized using ether and an abdominal incision was used to expose the liver. Then, 100 µL cell suspension was injected into the left lobe of liver, pressure was applied to the puncture point for 2–5 min until there was no active bleeding, and the abdominal cavity was closed. The mice were sacrificed by breaking their cervical vertebra after 8 weeks, and the livers and lungs were separated. Metastatic tumors were examined and made into paraffin sections for histopathological analysis. The study was approved by the Animal Care and Use Committee of Chongqing Medical University.

### Western blotting and Cycloheximide (CHX) Chase Assays

Total proteins were extracted from HCC tissue samples or cells using RIPA lysis buffer (Beyotime Institute of Biotechnology, Shanghai, China) containing protease inhibitors. The protein concentrations were determined using a BCA Protein Assay Kit (Beyotime Institute of Biotechnology), and proteins were separated on polyacrylamide gels and transferred to polyvinylidene fluoride (PVDF) membranes. Then, membranes were blocked with 5% skimmed milk for 2 h at room temperature and incubated with primary antibodies overnight at 4°C. After washing with TBST three times, the membranes were incubated with secondary antibodies conjugated to HRP (Cell Signaling Technology) for 2 h at room temperature. Finally, proteins were visualized using an ECL Plus kit (Bio-Rad, Hercules, CA, USA). The relative protein expression was analyzed using Quantity One software (Bio-Rad).

To examine the half-life of TGFR-1, Cycloheximide (CHX) Chase assay was performed. SK-Hep1-shRNA/USP4 cells or SK-Hep1-USP4 cells were treated with protein synthesis inhibitor CHX (100 µg/mL) for the indicated times. Then, proteins were extracted and Western blotting was performed.

### Co-immunoprecipitation (CO-IP) and ubiquitination assays

Cells were extracted with IP lysis buffer (Thermo Fisher Scientific, MA, USA) containing protease inhibitors. Protein A magnetic beads (Merck Millipore, Billerica, MA, USA) were added to the whole-cell lysates to remove non-specific proteins before immunoprecipitations. Pure lysates were immunoprecipitated with target antibodies overnight at 4°C. After washing with PBS, beads were added to the reactive lysates, and incubated for 2 hours at 4°C. Then, supernatants were discarded on a magnetic frame. Beads were suspended with loading buffer after washing with PBS twice and boiled at 100°C for 10 min. Finally, beads were removed on a magnetic frame to yield the immunoprecipitated protein complexes. western blotting was performed to analyze the protein complexes.

For ubiquitination assay, SK-Hep1cells were co-transfected with TGFR-1and shRNA/USP4, co-transfected with TGFR-1and USP4. Cells were treated with Proteasome inhibitor MG132(10uM) for 6h. Then, proteins were extracted and CO-IP assay was performed to detected ubiquitination of TGFR-1.

### Statistical analysis

Data are presented as means ± standard deviations (SDs). Each assay was performed three times in independent experiments. Statistical analyses were performed using SPSS (version 22.0). Student’s *t*-tests were used to compare two groups of independent samples. ANOVA was performed to compare multiple groups. Nonparametric tests were used to analyze the difference in USP4 expression between HCC and adjacent tissues. The relationship between USP4 expression and clinicopathological factors was assessed using Spearman correlation analysis. Kaplan-Meier survival analysis was used to investigate the prognostic relevance of USP4 in univariate analysis. The correlation between the expression of USP4 and other proteins was calculated using Spearman rank correlation. p < 0.05 was considered significant.

## Supplementary Material

Supplementary Table 1
