# Correction for: Ubiquitin-specific protease 4 promotes metastasis of hepatocellular carcinoma by increasing TGF-β signaling-induced epithelial-mesenchymal transition

**DOI:** 10.18632/aging.102010

**Published:** 2019-05-31

**Authors:** Chan Qiu, Yan Liu, Ying Mei, Min Zou, Zhibo Zhao, Mingxin Ye, Xiaoling Wu

**Affiliations:** 1 Department of Gastroenterology, the Second Affiliated Hospital of Chongqing Medical University, Chongqing 400010, P.R. China; 2 Department of Gastroenterology, the Fifth People’s Hospital of Chengdu, Chengdu, Sichuan 611130, P.R. China; 3 Department of Hepatobiliary Surgery, the Second Affiliated Hospital of Chongqing Medical University, Chongqing 400010, P.R. China; 4 Department of Hepatobiliary Surgery, the Affiliated Hospital of Southwest Medical University, Lu zhou, Sichuan 646000, P.R. China; * Equal contribution

Original article: Aging (Albany NY) 2018; 10:2783–2799

**This article has been corrected:** The authors submitted the wrong Figure 5 (panel F) from other experiment group in this study and requested to replace it. The correct Figure 5 is provided below. The authors declare that this correction does not change the results or conclusions of this paper. The authors sincerely apologize for this error.

**Figure 5:**
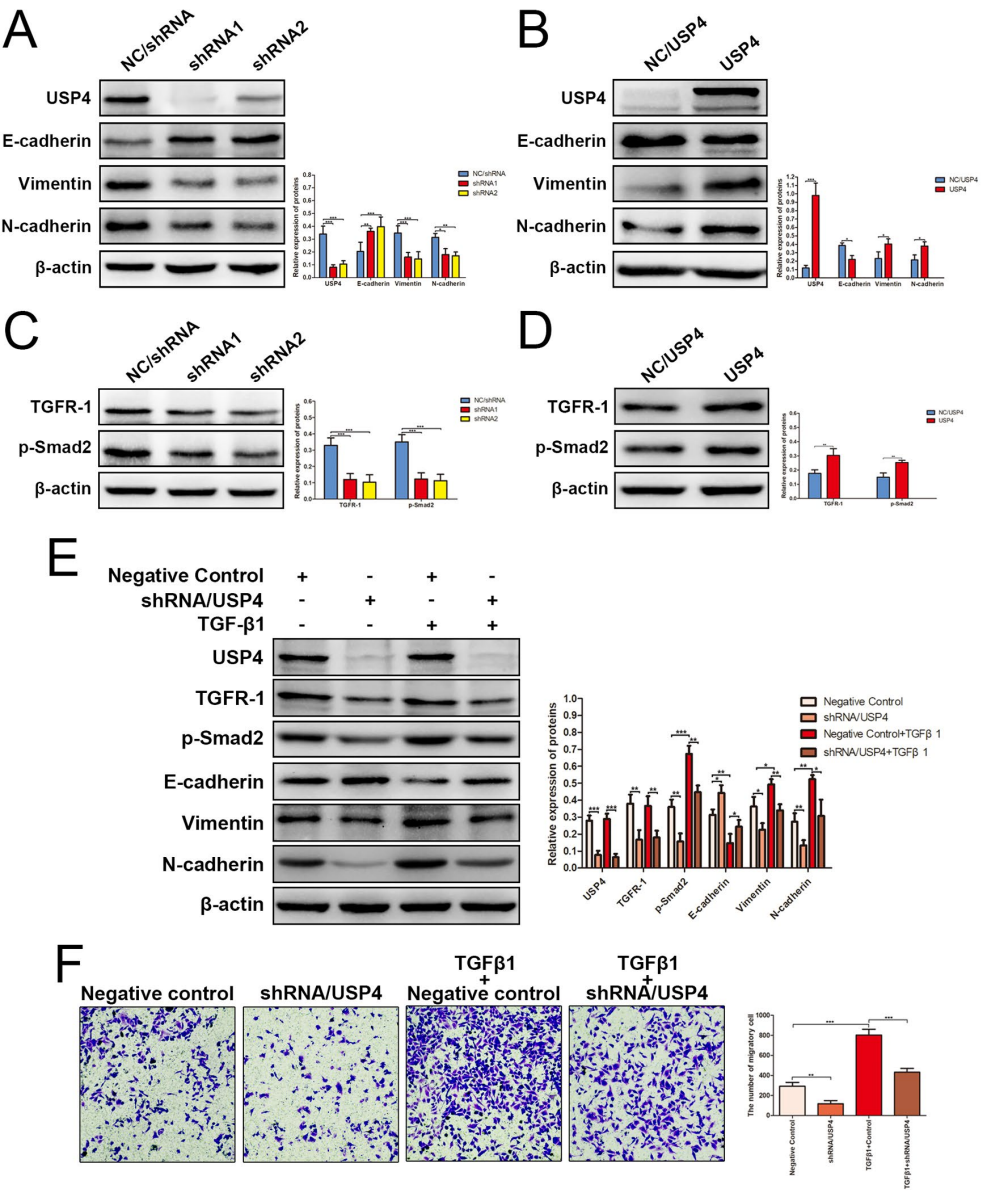
**USP4 activated TGF-β signaling pathway to induce epithelial-to-mesenchymal transition (EMT).** (**A**) The expression of EMT markers in SK-Hep1- shRNA/USP4 cells and in negative control cells (* P < 0.05, ** P < 0.01, *** P < 0.001). (**B**) The expression of EMT markers in HuH7-USP4 cells and in negative control cells (* P < 0.05, *** P < 0.001). (**C**) The expression of TGFR-1 and p-Smad2 in SK-Hep1- shRNA/USP4 cells and in negative control cells (*** P < 0.001). (**D**) The expression of TGFR-1 and p-Smad2 in HuH7-USP4 cells and in negative control cells (* P < 0.05, ** P < 0.01). (**E**) The expression of TGFR-1, p-Smad2 and EMT markers in SK-Hep1- shRNA/USP4 cells or in negative control cells, with TGF-β1(10ng/mL)treatment for 24h or without (* P < 0.05, ** P < 0.01, *** P < 0.001). (**F**) The migration ability of SK-Hep1-shRNA/USP4 cells or negative control cells, with TGF-β1 treatment or without, detected by Transwell migration assay (** P < 0.01, *** P < 0.001).

