# Supplementary material for: Ubiquitin-speciﬁc protease 4 promotes metastasis of hepatocellular carcinoma by increasing TGF-β signaling-induced epithelial-mesenchymal transition
Source: Aging (Albany NY). 2018 Oct 18;10(10):2783–99. doi: 10.18632/aging.101587 (PMC6224254; doi:10.18632/aging.101587)
Supplement: Supplementary Table 1 [file aging-10-101587-s001.docx]

| **Supplementary Table 1. Clinicopathological Characteristics of Patients with Hepatocellular Carcinoma (n=95).** | | |
| --- | --- | --- |
| **Characteristics** | **Value** | **%** |
| **Sex** |  |  |
| **Male** | **85** | **89.47** |
| **Female** | **10** | **10.52** |
| **Age** |  |  |
| **median(range), years** | **54(25~73)** |  |
| **Pathological grading** |  |  |
| **I** | **3** | **3.16** |
| **I~II** | **4** | **4.21** |
| **II** | **54** | **56.54** |
| **II~III** | **19** | **20.00** |
| **III** | **15** | **15.79** |
| **Toumor size** |  |  |
| **Range, cm** | **1*11*1~30*15*10** |  |
| **TNM** |  |  |
| **T1** | **12** | **12.63** |
| **T2** | **31** | **32.63** |
| **T3** | **41** | **43.16** |
| **T4** | **3** | **3.16** |
| **Unknown** | **8** | **8.42** |
| **N0** | **85** | **89.47** |
| **N1** | **1** | **1.05** |
| **Nx** | **1** | **1.05** |
| **Unknown** | **8** | **8.42** |
| **M0** | **86** | **90.52** |
| **M1** | **1** | **1.05** |
| **Mx** | **1** | **1.05** |
| **Unknown** | **7** | **7.37** |
| **AJCC clinical stage** |  |  |
| **I** | **12** | **12.63** |
| **II** | **31** | **32.63** |
| **III** | **41** | **43.16** |
| **III-IV** | **1** | **1.05** |
| **IVA** | **1** | **1.05** |
| **IVB** | **1** | **1.05** |
| **Unknown** | **8** | **8.42** |
| **Suvival status** |  |  |
| **Alive** | **34** | **35.79** |
| **Dead** | **61** | **64.21** |

www.aging-us.com 16 AGING
